# Acute Secondary Adrenal Insufficiency Misdiagnosed as Acute Cholecystitis

**DOI:** 10.1155/2021/8318747

**Published:** 2021-11-30

**Authors:** Moslem Sedaghattalab, Amir Hossein Doustimotlagh

**Affiliations:** ^1^Department of Internal Medicine, Yasuj University of Medical Sciences, Yasuj, Iran; ^2^Medicinal Plants Research Center, Yasuj University of Medical Sciences, Yasuj, Iran

## Abstract

Hypopituitarism refers to insufficient secretion of the pituitary hormones. Patients with acute adrenocorticotropic hormone (ACTH) deficiency may be presented with fatigue, dizziness, orthostatic hypotension, hypoglycemia, nausea, vomiting, or nonspecific abdominal pain. This study described an unusual case of hypopituitarism in a patient who presented with general abdominal pain, abdominal tenderness, nausea, vomiting, hypotension, and hypoglycemia. At first, the patient was admitted with the diagnosis of acute cholecystitis, but after treatment of hypopituitarism and adrenal insufficiency, his symptoms resolved completely, without the need for surgery. Hypopituitarism and secondary adrenal insufficiency should be considered in the differential diagnosis of the patients who present with acute abdomen, hypotension, or hypoglycemia.

## 1. Introduction

The pituitary gland is divided into anterior and posterior lobes. Anterior pituitary hormones include TSH (thyroid stimulating hormone), LH (luteinizing hormone), FSH (follicles-stimulating hormone), ACTH (adrenocorticotropic hormone), GH (growth hormone), and prolactin. ADH (antidiuretic hormone) is produced by the hypothalamus and travels to the posterior pituitary gland through the pituitary stalk [1]. Hypopituitarism refers to insufficient secretion of the aforementioned pituitary hormones. Diagnosis of hypopituitarism is confirmed via measurement of the levels of those hormones [2].

Causes of hypopituitarism include pituitary cell destruction (accounts for more than 95% of cases), by pituitary adenoma [3], hypothalamic tumors (meningiomas and craniopharyngiomas), surgical or radiation therapy of the pituitary adenoma, and infiltrative lesions such as histiocytosis and hereditary hemochromatosis [4]. Sheehan's syndrome (postpartum pituitary apoplexy) is a common cause of hypopituitarism in women in developing countries [5, 6]. Patients with ACTH deficiency may present with fatigue, dizziness, orthostatic hypotension, hypoglycemia, nausea, vomiting, or nonspecific abdominal pain [7, 8]. In this study, an unusual case of hypopituitarism and secondary adrenal insufficiency was described in a patient who presented with acute abdomen.

## 2. Case Report

A 70-year-old woman presented to emergency ward with general abdominal pain, nausea, and vomiting. She also had a history of hypothyroidism. Medication was levothyroxine. Her siblings had minor thalassemia.

**Figure 1:**
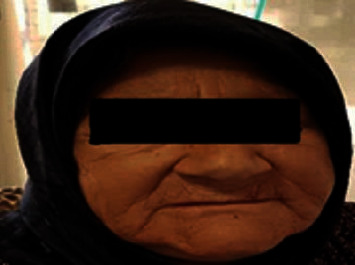
Alabaster-colored pale skin of patient seen in secondary adrenal insufficiency.

On examination, the body temperature, blood pressure, heart rate, respiratory rate, and oxygen saturation were 36°C, 80/60 mm Hg, 100 beats/min, 22 breaths/min, and 90%, respectively. The patient was pale (Figure 1). Abdominal examination revealed tenderness in right upper quadrant and positive Murphy's sign. All other examinations were normal. Body mass index was 23 (kg/m^2^).

The patient was admitted in general surgery ward with impression of acute cholecystitis, and internal medicine consult was requested for managing hypotension and hypoglycemia. The patient's history of hypothyroidism, abdominal pain, hypotension, and hypoglycemia suggested adrenal insufficiency as the main cause of her symptoms and signs.

The hemoglobin was 8 (g/dL), the platelet counts 160 (10^9^/L), and the white blood cell counts 8.2 (10^12^/L). The alanine aminotransferase was 13 (IU/L), aspartate aminotransferase 35 (IU/L), alkaline phosphatase 280 (IU/L), albumin 4.5 (g/dL), total bilirubin 2.7 (mg/dL), conjugated bilirubin 0.7 (mg/dL), erythrocyte sedimentation rate (ESR) 24.5 (mm/h), serum iron 165 (*μ*g/dL), total iron binding capacity (TIBC) 191 (*μ*g/dL), PH (potential of hydrogen) 7.25, CO_2_ (carbon dioxide) 30 (mm Hg), HCO_3_ (bicarbonate) 13 (mEq/L), sodium 144 (mEq/L), potassium 3.8 (mEq/L), blood sugar 40 (mg/dL), blood urea nitrogen (BUN) 28 (mg/dL), creatinine 1.7 (mg/dL), LH 0.5 (IU/L), FSH 0.3 (IU/L), cortisol 2 (*μ*g/dL), ACTH 0.2 (pg/L), and thyroid stimulating hormone (TSH) 2.1 (mU/L). Other laboratory tests, such as partial thromboplastin time, prothrombin time, and lipase, were normal.

Abdominal ultrasonography revealed gallstones and positive Murphy's sign. A computerized tomography (CT) scan of the chest and abdomen revealed linear atelectasis of right lower lobe of the lung and gallstone without any evidence of acute cholecystitis. The patient refused to do hypophysis MRI because of elevated creatinine and the risk of nephrogenic systemic fibrosis.

Treatment with hydrocortisone, normal saline, and dextrose water was initiated, and the patient's symptoms resolved completely without need to surgery.

Two months after initial presentation and treatment, our patient had no morbidity relating to acute abdomen, and treatment with hydrocortisone and levothyroxine is continuing. She had no any visual complaint before treatment and during follow-up time.

## 3. Discussion

ACTH deficiency leads to secondary adrenal insufficiency, in which cortisol secretion from adrenal gland reduces, but mineralocorticoid production is preserved; this is contrary to primary adrenal insufficiency, in which both cortisol and mineralocorticoid production are reduced. Secondary adrenal insufficiency is characterized by weakness, anorexia, fatigue, nausea, vomiting, and occasionally, hypoglycemia [9].

Patients with chronic adrenal insufficiency present with nonspecific symptoms, such as loss of energy and fatigue. Acute adrenal insufficiency is more frequently observed in primary adrenal insufficiency, and patients may present with hypotension, nausea, vomiting, fever, features of acute abdomen with abdominal tenderness, stupor, and coma. Adrenal crisis can be triggered by surgery or other stresses such as intercurrent illness [9].

In this study, an unusual case of hypopituitarism and secondary adrenal insufficiency was described in a patient who presented with general abdominal pain, abdominal tenderness, nausea, vomiting, hypotension, and hypoglycemia. She had metabolic acidosis and acute kidney injury due to hypotension and tissue hypoperfusion.

The history of hypothyroidism in addition to reduced gonadotropin levels and ACTH level suggested involvement of all axes of hypophysis.

At first, the patient was admitted in general surgery ward with diagnosis of acute cholecystitis, but after treatment with hydrocortisone, normal saline, and dextrose water, the patient's symptoms resolved completely without any need to surgery. Although unusual, hypopituitarism and secondary adrenal insufficiency should be considered in differential diagnosis of patients presenting with acute abdomen, hypotension, or hypoglycemia.

## 4. Conclusion

Acute adrenal insufficiency and features of acute abdomen more frequently observed in patients with primary adrenal insufficiency in which both cortisol and mineralocorticoid production are reduced; however, in this case, it happened in secondary adrenal insufficiency [9, 10]. Then, secondary adrenal insufficiency and hypopituitarism should be considered in the differential diagnosis of the patients who present with acute abdomen, hypotension, or hypoglycemia.

## Data Availability

The data used to support this study are included within the article.
